# Osteocytic HIF-1α Pathway Manipulates Bone Micro-structure and Remodeling via Regulating Osteocyte Terminal Differentiation

**DOI:** 10.3389/fcell.2021.721561

**Published:** 2022-01-18

**Authors:** Kaizhe Chen, Jian Zhao, Minglong Qiu, Lianfang Zhang, Kai Yang, Leilei Chang, Peng Jia, Jin Qi, Lianfu Deng, Changwei Li

**Affiliations:** ^1^ Department of Orthopedics, Shanghai Key Laboratory for Prevention and Treatment of Bone and Joint Diseases with Integrated Chinese-Western Medicine, Shanghai Institute of Traumatology and Orthopedics, Ruijin Hospital, Shanghai Jiaotong University School of Medicine, Shanghai, China; ^2^ Department of Orthopedics, The Central Hospital of Taian, Shandong, China; ^3^ Department of Orthopedics, The First Affiliated Hospital of Soochow University, Suzhou, China; ^4^ Department of Orthopedics, The Second Affiliated Hospital of Soochow University, Suzhou, China

**Keywords:** HIF-1α, osteocyte maturation, bone remodeling, bone material property, bone micro-structure

## Abstract

The activation of hypoxia-inducible factor 1α (HIF-1α) signaling has promising implications for the treatment of bone diseases such as osteoporosis and skeletal fractures. However, the effects of manipulating HIF-1α pathway on bone micro-structure and remodeling should be fully studied before the clinical application of therapeutics that interfere with the HIF-1α pathway. In this study, we found that osteocyte-specific HIF-1α pathway had critical role in manipulating bone mass accrual, bone material properties and micro-structures, including bone mineralization, bone collagen fiber formation, osteocyte/canalicular network, and bone remodeling. In addition, our results suggest that osteocyte-specific HIF-1α pathway regulates bone micro-structure and remodeling via impairing osteocyte differentiation and maturation.

## Introduction

Hypoxia-inducible factor (HIF) α is a major transcriptional regulator of adaptive responses to low oxygen levels (Zuo et al., 2015). The HIF protein family comprises three α subunits: HIF-1α, HIF-2α, and HIF-3α. HIF-α is an oxygen-labile protein that forms a heterodimeric complex with the HIF-β subunit, which is constitutively expressed (Wang et al., 2007). Under normoxic conditions, HIF-α subunits are proline-hydroxylated by proline hydroxylase domain proteins (PHDs). Proline hydroxylation targets HIF-α subunits for ubiquitination by the E3 ligase complex von Hippel-Lindau (VHL) and ultimately, for degradation by the 26S proteasome. Under hypoxic conditions, the prolyl hydroxylation of HIF-α is inhibited, causing HIF-α to accumulate in the nucleus where it heterodimerizes with HIF-β subunits and transactivates HIF-responsive genes (Loots et al., 2018).

The VHL/HIF system is highly active within bone tissue. The inactivation of HIF-1α causes a marked decrease in trabecular bone volume, reduces the rate of bone formation, and alters the cortical bone architecture (Shomento et al., 2010). On the other hand, the deletion of PHD or VHL stabilizes HIF-1α, which then causes an increase in bone mass by regulating the bone formation activity of osteoblasts and the bone resorption activity of osteoclasts (Zhang et al., 2014; Wu et al., 2015; Kang et al., 2017; Loots et al., 2018). Furthermore, activating the HIF-1α pathway with hypoxia-mimicking agents such as deferoxamine and dimethyloxalylglycine can decrease the bone loss associated with ovariectomy-induced osteoporosis (Zhao et al., 2012; Peng et al., 2014), prevent ultra-high-molecular-weight polyethylene-induced osteolysis (Kang et al., 2016), and facilitate the healing of osteoporotic bone defects (Jia et al., 2016). Therefore, the modification of VHL/HIF signaling may be a promising treatment for bone diseases such as osteoporosis and skeletal fractures.

Although increasing HIF-1α pathway activation appears to have beneficial effects, the degree of HIF-1α activation must be fine-tuned to avoid disrupting bone homeostasis (Loots et al., 2018). Constitutive activation of HIF-1α via VHL deletion or the combined inactivation of PHD1, PHD2, and PHD3 leads to polycythemia and excessive bone accumulation (Wang et al., 2007; Wu et al., 2015). In addition, we previously found that constitutive activation of the HIF-1α pathway in mature osteoblasts using the osteocalcin (OC)-Cre promoter system disrupts the integrity of the osteocyte/canalicular network (Zuo et al., 2015). Since Cre enzyme can be inherited by osteocytes from their modified osteoblast derivatives (Chen et al., 2015), which leads to HIF-1α activation in osteocytes. And activation of the osteocytic HIF-1α pathway via VHL deletion causes an excessive bone mass phenotype (Loots et al., 2018). Therefore, the manipulating effect of HIF-1α on bone material properties and micro-structures as well as bone remodeling should be fully studied before the clinical application of therapeutics that interfere with the HIF-1α signaling pathway.

In this study, we deleted *Vhl* in mouse mineralizing osteocytes to generate mice in which the HIF-1α pathway was constitutively activated, which resulted in excessive immature bone accumulation with impaired bone material properties and micro-structures, including abnormal mineralization, disrupted collagen fiber formation, and an impaired osteocyte/canalicular network, which may result from impaired osteocyte terminal differentiation. Furthermore, the impaired osteocyte maturation caused by CA- HIF-1α in mineralizing osteocyte facilitated osteogenesis in mesenchymal stem cells (MSCs) and impaired osteoclastic differentiation of bone marrow monocytes (BMMs), which resulted in disrupted bone remodeling and may be responsible for the abnormal bone formation observed in osteocyte-specific *Vhl* deficient mice.

## Materials and Methods

### Mice

*Vhl* ^*fl/fl*^ *-Dmp-1-Cre* ^*+*^ mice were generated by intercrossing *Vhl* ^*fl/fl*^ mice with *Dmp1*-cre transgenic mice; for this strain, *Vhl* ^*fl/fl*^ *-Dmp-1-Cre* ^*−/−*^ (referred to as *Vhl* ^*fl/fl*^) littermates were used as the controls. *Vhl* ^*fl/fl*^ (C57/B6) mice were kindly provided by Dr. Thomas L. Clemens (Department of Orthopedic Surgery, John Hopkins University School of Medicine, Baltimore, MD). *Dmp-1-Cre* mice were purchased from the Jackson Laboratory (FVB/N, JAX Stock #023047; Sacramento, CA) and backcrossed into the C57/BL6J background over at least four generations to ensure that all mice were maintained within a C57BL/6 background. All procedures involving mice were approved by the Shanghai Jiaotong University Animal Study Committee and carried out in accordance with the institutional guidelines for the humane use and care of laboratory animals.

### Micro-CT Analysis

CT scans of mouse femurs were performed using a high-resolution SkyScan1172 Micro-CT machine (Bruker, Belgium) with the parameters set to 100 kVp, 100 Ma, and 10.0 μm/pixel. Serial tomographs were reconstructed from raw data using Conebeam reconstruction software (NRecon; Bruker), then used to compute trabecular and cortical parameters from the metaphyseal and mid-diaphyseal areas. All measurements were performed according to the guidelines of the American Society for Bone and Mineral Research.

### Histology and Histochemical Staining

For H&E, Safranin O-Fast green, toluidine blue, Sirius red, and TRAP staining, bones were fixed in 4% paraformaldehyde overnight at 4°C, decalcified for 2–4 weeks in 0.25 M ethylenediaminetetraacetic acid (EDTA) at pH 7.4, and then embedded in paraffin. Sections were stained with H&E for osteoarchitectural analyses. Briefly, paraffin sections were stained with hematoxylin for 5 min, followed by staining with eosin for 30 s, then the osteoblast and osteocyte parameters were measured. Safranin O-Fast green was applied to sections to stain acid mucopolysaccharides and collagen fibers in the bone matrix. The collagen composition after Sirius red staining was further visualized under polarized light. To visualize osteoclasts, a leukocyte acid phosphatase kit (Sigma, St. Louis, MO) was applied to paraffin sections to stain cells exhibiting TRAP activity. Osteoblast and osteocyte numbers and surface areas were quantified using H&E-stained sections. Osteoblast and osteoclast surface areas were expressed relative to the total bone surface area.

### Immunohistochemical and Immunofluorescence Analyses

Paraffin-embedded bone tissues were used for the immunohistochemical analysis of HIF-1α and RANKL expression. Briefly, paraffin sections were de-waxed, rehydrated, incubated with antigen retrieval solution, and then washed in tris buffered saline (TBS). Endogenous peroxidase activity was blocked by immersing the sections in methanol with 3% hydrogen peroxide (v/v) for 10 min. Unspecific antibody binding was blocked by incubating the sections in TBS supplemented with 2% bovine serum albumin (w/v) for 30 min. Subsequently, sections were incubated overnight with a primary antibody against HIF-1α (NB100-105, 1/50 dilution; Novus, Centennial, CO) or RANKL (AB45039, 1/100 dilution; Abcam, United Kingdom). The slides were then exposed to horseradish peroxidase (HRP)-conjugated streptavidin for 30 min, and antibody binding was visualized based on HRP activity with the colored substrate diaminobenzidine (DAB Kit; Invitrogen, Carlsbad, CA). Then, the slides were counterstained with hematoxylin.

To examine the expression of E11, DMP-1, and SOST via immunofluorescence staining, paraffin sections were de-waxed, rehydrated, and permeabilized with 0.5% Triton X-100, followed by the blocking of non-specific antibodies. The bone tissue was then incubated with anti-E11 monoclonal hamster antibody (MA5-16113, 5 μg/ml; Thermo, Waltham, MA), anti-DMP-1 polyclonal sheep antibody (AF4386, 10 μg/ml; R&D), or anti-SOST polyclonal rabbit antibody (AB63097, 1/50 dilution) at 4°C overnight. Next, the sections were incubated with secondary antibodies at room temperature for 30 min. The secondary antibodies used were hamster immunoglobulin G (IgG) (H + L; Alexa Fluor 488, 1/1,000 dilution; Invitrogen), sheep IgG (H + L; Alexa Fluor 647, 1/1,000 dilution; Invitrogen), and rabbit IgG (H + L; Alexa Fluor 555, 1/1,000 dilution; Invitrogen).

The paraffin sections were labeled with phalloidin (Alexa Fluor 594, 5 μg/ml; Life Technologies, Thermo) and 4, 6-diamidino-2-phenyindole dilactate (10 μg/ml; Life Technologies) to stain F-actin to indirectly visualize the cytoskeleton. The sections were then examined under laser scanning confocal microscopy.

### Bone Dynamic Histomorphology and Undecalcified Bone Tissue Sections

To analyze dynamic bone parameters, 24-week-old *Vhl* ^*fl/fl*^ *-Dmp-1-Cre* ^*+*^ mice and their respective littermate controls were labelled at 10 days (calcein, 30 mg/kg; Sigma; intraperitoneal injection) and 3 days (alizarin, 30 mg/kg; Sigma; intraperitoneal injection) prior to sacrifice. Undecalcified tissue sections of the long bones of lower limbs were prepared by fixing the samples in 70% ethanol at 4°C for 48 h, then the specimens were dehydrated in acetone and embedded in methyl methacrylate resin. From these, 50-µm femur sections were cut using a microtome (EXAKT, Oklahoma City, OK) and visualized under laser scanning confocal microscopy. All measurements were performed using triplicate sections.

### Electron Microscopy Analysis

Acid etching of resin-casted bone samples was performed to examine the lacuna-canalicular network with SEM. Briefly, resin-casted bone sections were immersed in 9% phosphoric acid solution for 30 s, followed by a short rinse in deionized water (1–2 s). The samples were then exposed to 5% sodium hypochlorite for 5 min, rinsed in deionized water, and air dried naturally at room temperature. Subsequently, the samples were coated with a 20-nm gold layer and examined via SEM (Zeiss, Germany). To further examine the ultrastructure of osteocytes, fresh femurs were obtained from *Vhl* ^*fl/fl*^ *-Dmp-1-Cre* ^*+*^ mice, and their corresponding littermate controls for TEM. Femur samples were pre-fixed in 2.5% glutaraldehyde at 4°C overnight and then decalcified in 0.25 M EDTA at pH 7.4 for 2–4 weeks. Next, the diaphysis was cut into small cubes, post-fixed in 1% osmium tetroxide, dehydrated in acetone, and then embedded in Epon. TEM (Hitachi, Japan) was used to examine the ultrathin sections.

### Bone Biomechanical Testing

To evaluate the long bone strength, the left femurs of 24-week-old mice were separated and wrapped in gauze soaked in 0.9% saline solution prior to performing a three-point bending test. A material test machine (5500R; Instron, Norwood, MA) was used to test each femur to failure; each sample completed the test within 1 h. The femurs were positioned horizontally with the anterior surface upwards, centered on the holders with a 10-mm distance between each sample. A load was applied perpendicular to the midpoint of the femur constantly at a displacement rate of 0.5 mm/s. A load-displacement curve was then plotted and used to calculate the mechanical parameters of each femur sample (elastic modulus, ultimate stress, and bending stiffness).

Nanoindentation was used to further evaluate the microscopic biomechanical characteristics of mouse long bones. First, resin-casted femur specimens were ground by hand using a sequence of silicon-carbon abrasive papers of decreasing abrasiveness (P1200, P2500, and P4000-grit) under continuous water cooling until the specimens reached a thickness of 50 µm. The specimens were further polished using a special flannel and polishing liquid comprising aluminum oxide grains and triple-distilled water until the specimens were 30–40 µm thick. The slices were smoothly bonded to the test bench using a hot dryer, then 10 test points within the trabecular bone area below the metaphysis were randomly selected. The parameters were set as follows: surface approach velocity, 10 nm/s; depth limit, 2000 nm; strain rate target, 0.05 s^−1^; harmonic displacement target, 2 nm; frequency target, 45 Hz; Poisson’s ratio, 0.3. The data used to calculate the elastic modulus and nanohardness were obtained over a depth range of 100–2000 nm.

### IDG-SW3 Cell Culture

Immortalized IDG-SW3 cells were kindly gifted by Dr. Lynda Bonewald (Indiana University). As previously described (Woo et al., 2011), the IDG-SW3 cells were cultured on type I collagen-coated plates and seeded at 1 × 10^5^ cells/mL for the experiments. Cells were cultured in α-minimum essential medium (α-MEM) with 10% heat-inactivated fetal bovine serum (FBS), 100 U/mL penicillin/streptomycin (P/S), and 50 U/mL interferon-gamma (IFN-γ) for cell expansion at 33°C under 5% CO_2_. Cells were differentiated at 37°C in differentiation medium, which comprised α-MEM, 10% FBS, 100 U/mL P/S, 50 μg/ml ascorbic acid, and 4 mM β-glycerophosphate. Generally, the cells differentiated into mature osteocytes after 4 weeks under differentiation conditions, and the mature cells were used in subsequent experiments.

To mimic an *in vivo* environment, IDG-SW3 cells were embedded in 3D collagen gel. The cell embedding solution was prepared by mixing 2.4 mg/ml neutralized collagen solution with the same volume of Matrigel basement membrane matrix as previously described (Kurata et al., 2006), IDG-SW3 cell proliferation was inhibited, and the cells were suspended in the collagen/Matrigel mixture at a density of 2 × 10^5^ cells/mL. The cell-containing gel mixture was planted in a culture plate and then incubated at 37°C for 30 min to solidify the mixture. Differentiation medium was then added to the culture plates, which were incubated at 37°C to trigger differentiation.

### siRNA Interference

To suppress endogenous *Vhl* expression, adenoviruses carrying siRNA against the 3ʹ untranslated region of mouse VHL or HIF-1α mRNA were constructed using a pAd/Easy-U6-CMV-EGFP vector (HanBio, China). siRNA oligonucleotides of *Vhl* and *Hif-1α* were shown in Table 1. A mixture of three siRNA adenovirus targeting *Vhl* or *Hif-1α* was used to transfected IDG-SW3 cells when they reached 80% confluence. Knockdown efficiency was tested by RT–qPCR.

**TABLE 1:** siRNA oligonucleotides.

	Sense (5‘-3’)	Antisense (5′-3′)
*Vhl* siRNA-1	CCGCAUAUGUAGGGCAUAUdTdT	AUAUGCCCUACAUAUGCGGdTdT
*Vhl* siRNA-2	GUUAACCAAACGGAGCUGUdTdT	ACAGCUCCGUUUGGUUAACdTdT
*Vhl* siRNA-3	CAUUGCCAGUGUAUACCCUdTdT	AGGGUAUACACUGGCAAUGdTdT
*Hif-1α* siRNA-1	GCUGACCAGUUACGAUUGUdTdT	ACAAUCGUAACUGGUCAGCdTdT
*Hif-1α* siRNA-2	CUGAUAACGUGAACAAAUAdTdT	UAUUUGUUCACGUUAUCAGdTdT
*Hif-1α* siRNA-3	GACACAGCCUCGAUAUGAAdTdT	UUCAUAUCGAGGCUGUGUCdTdT
Negative control	UUC​UCC​GAA​CGU​GUC​ACG​UAA	UUA​CGU​GAC​ACG​UUC​GGA​GAA

### Cell Co-culture Experiments

To investigate the biological effects of osteocytes on surrounding cells involved in bone remodeling, IDG-SW3 cells were co-cultured with BMMs or BMSCs with or without cell-to-cell contact. Primary mouse BMMs and BMSCs were isolated from the femur and tibia as previously described (Kang et al., 2017). To perform co-culture experiments with cell-to-cell contact, BMMs or BMSCs were directly seeded (1 × 10^5^ cells per well in a 12-well plate) onto IDG-SW3 cells, which had been subjected to 4-weeks osteogenic differentiation in 2D or 3D culture.

To perform co-culture experiments without cell-to-cell contact, IDG-SW3 were seeded onto 12-well Transwell inserts (1-μm pore size) in a 2D plane or in 3D gel, and then BMMs or BMSCs were planked at the top of the Transwell chamber or at the bottom of the culture plate, respectively. Consequently, BMMs and BMSCs within the Transwell plates could directly communicate with IDG-SW3 cells, but only through secreted cytokines. During the co-culture period, α-MEM was supplemented with 10% FBS and 100 U/mL P/S only, without the addition of osteogenic factors such as ascorbic acid and β-glycerophosphate.

### Cytohistochemical Fluorescence Staining

The cytoskeletal morphology of differentiating IDG-SW3 cells was examined by labelling cellular F-actin with phalloidin. Differentiation was induced in IDG-SW3 cells, which were labelled and examined using confocal microscopy on day 1 and at weeks 1, 2, and 3 of differentiation. In addition, cells were stained with Alizarin red (2% [w/v] Alizarin red S; Sigma) to visualize mineralization deposits formed by IDG-SW3 cells following disruption of the HIF-1α pathway by *Hif-1α*-siRNA or *Vhl*-siRNA.

### Enzyme Activity Detection

Osteoclast formation was detected in 7-days co-cultures of BMMs and IDG-SW3 cells using a TRAP staining kit (Sigma), and TRAP-positive areas were visualized with a stereomicroscope. An ALP staining kit (Beyotime, China) was used to measure the ALP level in BMSCs following co-culture with IDG-SW3 cells for 7 days. The manufacturers’ instructions were followed for both assays.

### Serum/Supernatant Enzyme-Linked Immunosorbent Assay

The sera of 24-week-old *Vhl* ^*fl/fl*^ *-Dmp-1-Cre* ^*+*^ mice, and their corresponding littermate controls were collected for cytokine content measurements. Serum contents of P1NP and CTX-1, cytokines indicating bone formation and bone resorption, respectively, were measured using ELISA (Novus). To measure RANKL secretion levels in IDG-SW3 and BMM co-cultures, supernatants from the upper layer of co-culture Transwell plates and the lower layer of co-culture Petri dishes of the co-cultures were collected for analysis via RANKL-specific ELISA (R&D, Minneapolis, MN). To examine SOST secretion levels in IDG-SW3 and BMSC co-cultures, the supernatants were harvested, centrifuged to remove cell impurities, and then subjected to SOST-specific ELISA (R&D).

### Quantitative Real-Time-PCR

Total RNA from IDG-SW3 cells was extracted using TRIzol reagent (Invitrogen) as previously reported. cDNA was synthesized using 1 μg of RNA and a RevertAid First Strand cDNA Synthesis kit (TaKaRa, Japan). qRT-PCR was then performed to amplify the cDNA using a SYBR Premix Ex Tag kit (TaKaRa) on an ABI 7500 Sequencing Detection System (Applied Biosystems, Foster City, CA). The primer sequences used in this study are listed in Table 2.

**TABLE 2:** Gene primer sequences used for real-time PCR.

Species	Genes	Primer sequences (5’→3′)	GenBank accession no
Mouse	β-Actin	GGT​TGT​CTC​CTG​CGA​CTT​CA	NC_000071.6
		TGG​TCC​AGG​GTT​TCT​TAC​TCC	
Mouse	Pdpn (E11)	GGA​CCG​TGC​CAG​TGT​TGT​TCT​G	NM_010329.3
		ACC​ATG​CCG​TCT​CCT​GTA​CCT​G	
Mouse	Phex	TGG​CTG​AGA​CAC​AAT​GTT​GAC​CTC	NM_011077.2
		GCC​TCA​AGA​TGT​GGA​GCA​GTG​G	
Mouse	Dmp-1	CTG​AGT​TCG​ATG​ATG​AAG​GGA​T	NM_001359013.1
		CCT​TTA​GAT​TCT​TCC​GAC​CTG​A	
Mouse	Sost	CTG​AGA​ACA​ACC​AGA​CCA​TGA​A	NM_024449.6
		CTG​TAC​TCG​GAC​ACA​TCT​TTG​G	
Mouse	Alp	TCA​TTC​CCA​CGT​TTT​CAC​ATT​C	NM_001287172.1
		GTT​GTT​GTG​AGC​GTA​ATC​TAC​C	
Mouse	OC (Bglap)	AAG​CCT​TCA​TGT​CCA​AGC​AGG​AG	NM_007541.3
		CGG​TCT​TCA​AGC​CAT​ACT​GGT​CTG	
Mouse	Runx2	CCT​TCA​AGG​TTG​TAG​CCC​TC	NM_001146038.2
		GGA​GTA​GTT​CTC​ATC​ATT​CCC​G	

### Western Blot Analyses

We performed Western blot analyses to examine the expression of osteocyte-derived RANKL, as described previously. Briefly, 15 μg of plasma proteins was extracted from IDG-SW3 cells and then separated using 10% sodium dodecyl sulfate-polyacrylamide gel electrophoresis. The proteins were electroblotted onto polyvinylidene difluoride membranes (0.45 mm; Millipore, Bedford, MA, United States), which were then blocked using 5% non-fat dry milk in TBS with Tween 20 for 1 h. The membranes were then incubated at 4 °C overnight with an anti-RANKL antibody (AB45039, 1/1,000 dilution; Abcam), followed by incubation with HRP-conjugated secondary antibodies (1/5,000 dilution; Jackson Laboratory) at room temperature for 1 h. An enhanced chemiluminescence assay (Thermo Scientific, Pierce, Rockford, IL, United States) was then applied to visualize antigen–antibody complexes.

### Von Kossa Staining

Undecalcified sections of the femurs of 8-week-old *Vhl* ^*fl/fl*^ *-Dmp-1-Cre* ^*+*^ male mice and their littermate controls were prepared for Von Kossa staining as previously reported (Stegen et al., 2018). Briefly, sections were treated with 5% AgNO_3_ for 1h, resulting in silver deposits at sites of calcium incorporation. The staining was fixed with 5% Na_2_S_2_O_3_ and sections were counterstained with haematoxylin. The mineralized area of the bone was dyed black and measured with OsteoMeasure software. The von Kossa/bone surface (%) was used to represent the proportion of mineralized area to bone surface.

### Statistical Analyses

Data were collected from three or more independent experiments and are presented as the mean ± standard error of the mean. We used two-tailed t-tests to determine significances between two groups. We did analyses of multiple groups by one- or two-way ANOVA with Bonferroni post-test of GraphPad prism version 7.0. *p* < 0.05 was considered to indicate significant differences.

## Results

### Osteocyte-specific HIF-1α Orchestrates Bone Mass Accrual and Bone Material Properties

**FIGURE 1:**
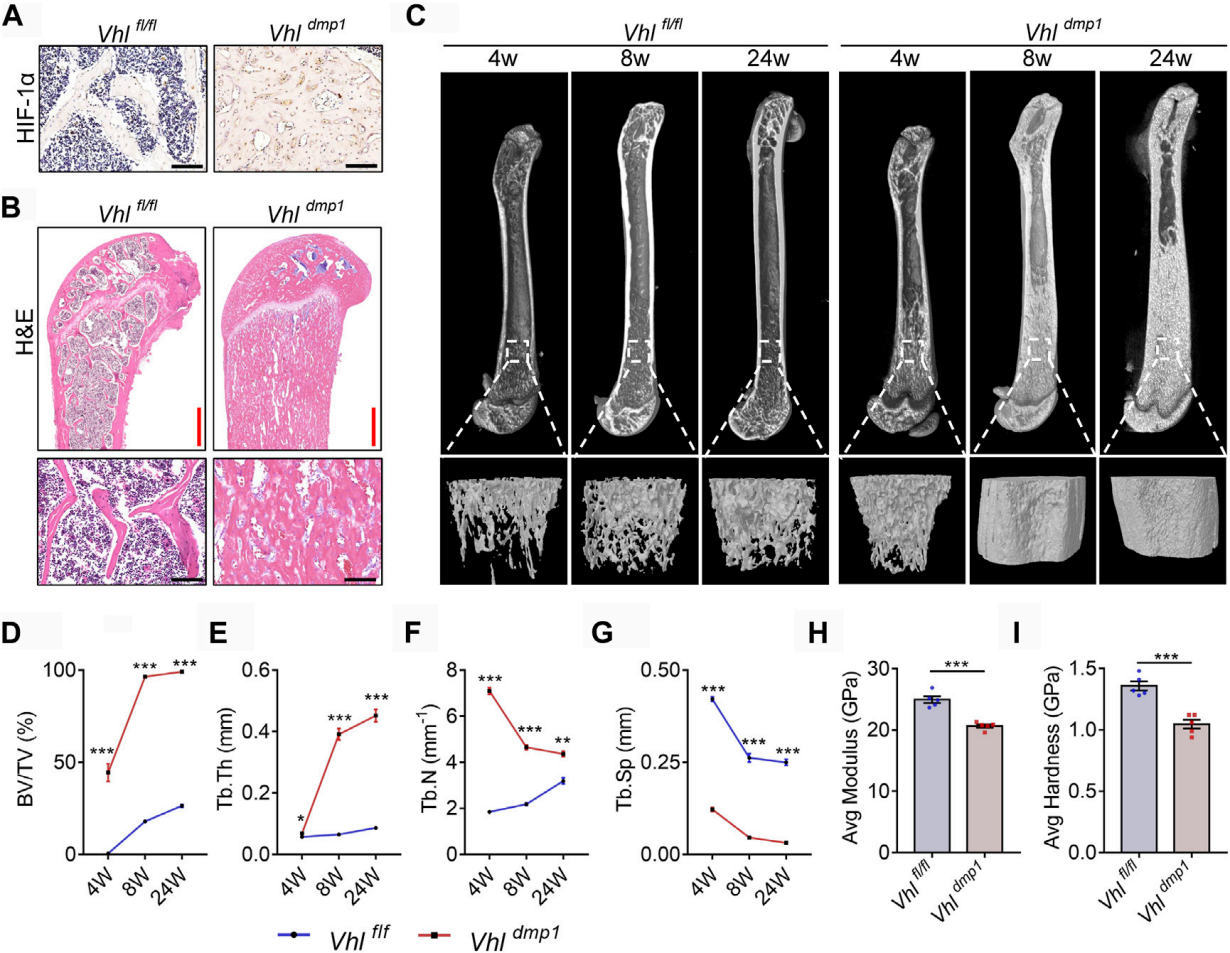
Continuous activation of HIF-1α in osteocytes causes excessive bone formation and impairs bone material properties. **(A)** Representative images of immunohistochemical staining of HIF-1α in the femurs of 2-month-old *Vhl* ^*f/f*^ *-Dmp-1-Cre* ^*+*^ and *Vhl* ^*f/f*^ *-mp-1-Cre* ^*−/−*^ male mice. Scale bars represent 100 μm. **(B)** Representative images showing hematoxylin and eosin (H&E) staining of femurs of 2-month-old *Vhl* ^*f/f*^ *-Dmp-1-Cre* ^*+*^ and *Vhl* ^*f/f*^ *-Dmp-1-Cre* ^*−/−*^ male mice. Red and black scale bars represent 500 and 100 μm, respectively. **(C)** Representative micro-computed tomography (micro-CT) images of femurs and the corresponding trabecular bone from the femoral metaphysis of 4-, 8-, and 24-week-old *Vhl* ^*f/f*^ *-Dmp-1-Cre* ^*+*^ and *Vhl* ^*f/f*^ *-Dmp-1-Cre* ^*−/−*^ male mice. **(D–G)** The trabecular bone volume (BV/TV) **(D)**, trabecular number (Tb.N) **(E)**, trabecular separation (Tb.Sp) **(F)**, and trabecular thickness (Tb.Th) **(G)** parameters of 4-, 8-, and 24-week-old *Vhl* ^*f/f*^ *-Dmp-1-Cre* ^*+*^ and *Vhl* ^*f/f*^ *-Dmp-1-Cre* ^*−/−*^ male mice were determined via micro-CT analysis. **(H,I)** Elastic modulus **(H)** and nanohardness **(I)** of the trabecular bone of 2-month-old *Vhl* ^*f/f*^ *-Dmp-1-Cre* ^*+*^ and *Vhl* ^*f/f*^ *-Dmp-1-Cre* ^*−/−*^ male mice, as determined by nanoindentation. *p < 0.05, **p < 0.01, ***p < 0.001. *p*-values were determined using two-way ANOVA in **(D–G)** and two-tailed *t*-tests in **(H,I)**.

To investigate the role of the HIF-1α pathway activation in bone material properties and micro-structures as well as bone remodeling, we generated mice in which the HIF-1α pathway in osteocytes was constitutively activated (CA-HIF-1α) by crossing *Vhl-flox* mice with *dentin matrix protein 1 (Dmp-1)-Cre* transgenic mice (Loots et al., 2018). Consistent with previous reports (Loots et al., 2018), osteocyte-specific *Vhl* deletion caused increased level of HIF-1α and an increase in cancellous bone mass (Figure 1A, Supplementary Figure S1). Hematoxylin and eosin (H&E) staining revealed that almost all the bone marrow spaces in the long bones of CA-HIF-1α mice were filled with cancellous bone (Figure 1B). Micro computed tomography (micro-CT) analyses revealed an imbalance in the trabecular bone micro-structures in the femur metaphyses of CA-HIF-1α mice (Figure 1C), as indicated by a dramatic increase in the trabecular bone volume (BV/TV), trabecular thickness (Tb.Th), and trabecular number (Tb.N) and a decrease in the trabecular spacing (Tb.Sp) (Figures 1D–G). Contrary to *Vhl* deletion, osteocyte-specific *Hif-1α* deletion leaded to decreased bone mass (Supplementary Figures S2A,B). Furthermore, the difference of bone mass between *Hif-1α* ^−/-^ mice and their WT controls was increased significantly with age increasing, as demonstrated by micro-CT analysis (Supplementary Figures S2C–G).

Examination of the surface mechanical properties of resin-embedded trabecular bone via nanoindentation revealed that the mechanical properties were also impaired in CA-HIF-1α mouse femurs; the elastic modulus and nanohardness of the trabecular bone were lower in CA-HIF-1α mice compared to the control mice (Figures 1H,I). These results suggest that the bone material properties of CA-HIF-1α mice were impaired.

### Osteocyte-specific HIF-1α Orchestrates Bone Mineralization and Bone Collagen Fiber Formation

**FIGURE 2:**
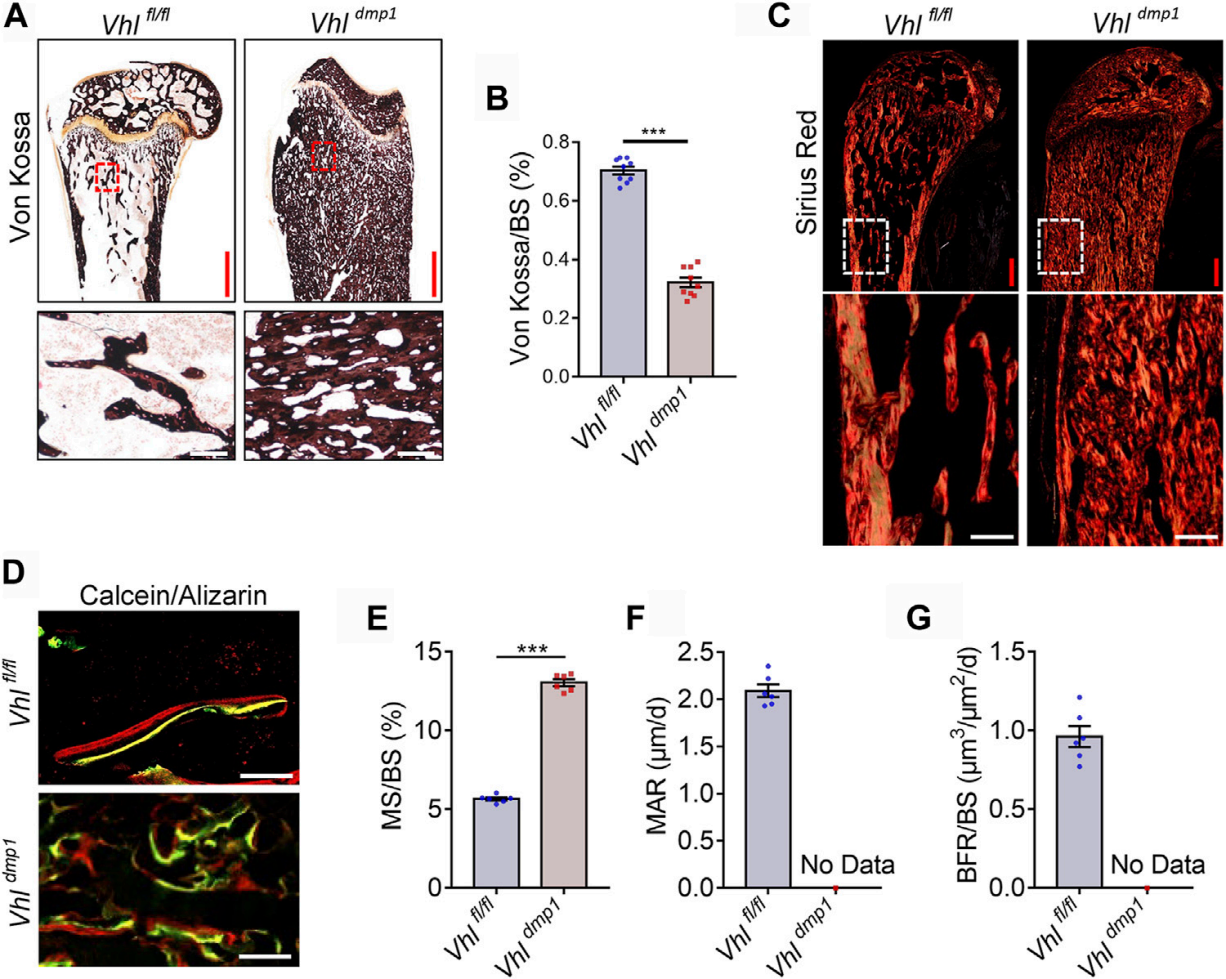
Constitutive activation of HIF-1α in osteocytes leads to abnormal mineralization and disrupted collagen fiber formation in bone. **(A,B)** Von Kossa staining and bar graph showing the proportional Von Kossa-positive surface area relative to total bone surface area (Von Kossa/BS) in the femurs of 2-month-old *Vhl* ^*f/f*^ *-Dmp-1-Cre* ^*+*^ and *Vhl* ^*f/f*^ *-Dmp-1-Cre* ^*−/−*^ male mice. Red and white scale bars represent 500 and 100 μm, respectively. **(C)** Sirius Red staining of the femurs of 2-month-old *Vhl* ^*f/f*^ *-Dmp-1-Cre* ^*+*^ and *Vhl* ^*f/f*^ *-Dmp-1-Cre* ^*−/−*^ male mice. Red and white scale bars represent 500 and 100 μm, respectively. **(D–G)** Representative images of double calcein/alizarin labeling of the femurs of 2-month-old Vhl^f/f^-*Dmp-1-Cre* ^*+*^ and *Vhl* ^*f/f*^ *-Dmp-1-Cre* ^*−/−*^ male mice **(D)** used to calculate the proportional mineral surface area over total bone surface area (MS/BS) **(E)**, mineral apposition rate (MAR) **(F)**, and bone formation rate per unit bone surface (BFR/BS) **(G)**. Scale bars represent 100 μm ***p < 0.001. *p*-values were determined using two-tailed *t*-tests.

The material properties of bone are affected by disruptions in mineralization and the collagen network (Albert et al., 2013). Von Kossa staining of undecalcified tibial sections showed that the mineralization level in trabecular bone was significantly lower in CA-HIF-1α mice compared to control mice (Figures 2A,B). Furthermore, staining with Sirius Red revealed that osteocytic CA-HIF-1α caused a significant increase in bone collagen, which filled most of the bone marrow space (Figure 2C). However, unlike the ordered collagen arrangement in the controls, collagen fibers were disorganized in the CA-HIF-1α mice (Figure 2C). Double calcein/alizarin labeling also revealed disordered new bone formation in the CA-HIF-1α mice (Figures 2D−G).

Contrary to *Vhl* deletion, osteocyte-specific *Hif-1α* deletion resulted in facilitated bone mineralization in osteocytic *Hif-1α* ^−/-^ mice (Supplementary Figures S3A,B). In addition, staining with Sirius Red revealed that osteocytic *Hif-1α* deficiency caused a significant decrease in bone collagen accumulation (Supplementary Figure S3C). Double calcein/alizarin labeling revealed that mice with osteocytic *Hif-1α* deletion showed lower dynamic bone formation rates (BFRs) than controls (Supplementary Figures S3D,E).

### Osteocyte-specific HIF-1α Regulates Osteocyte Differentiation and Maturation *in vivo*

**FIGURE 3:**
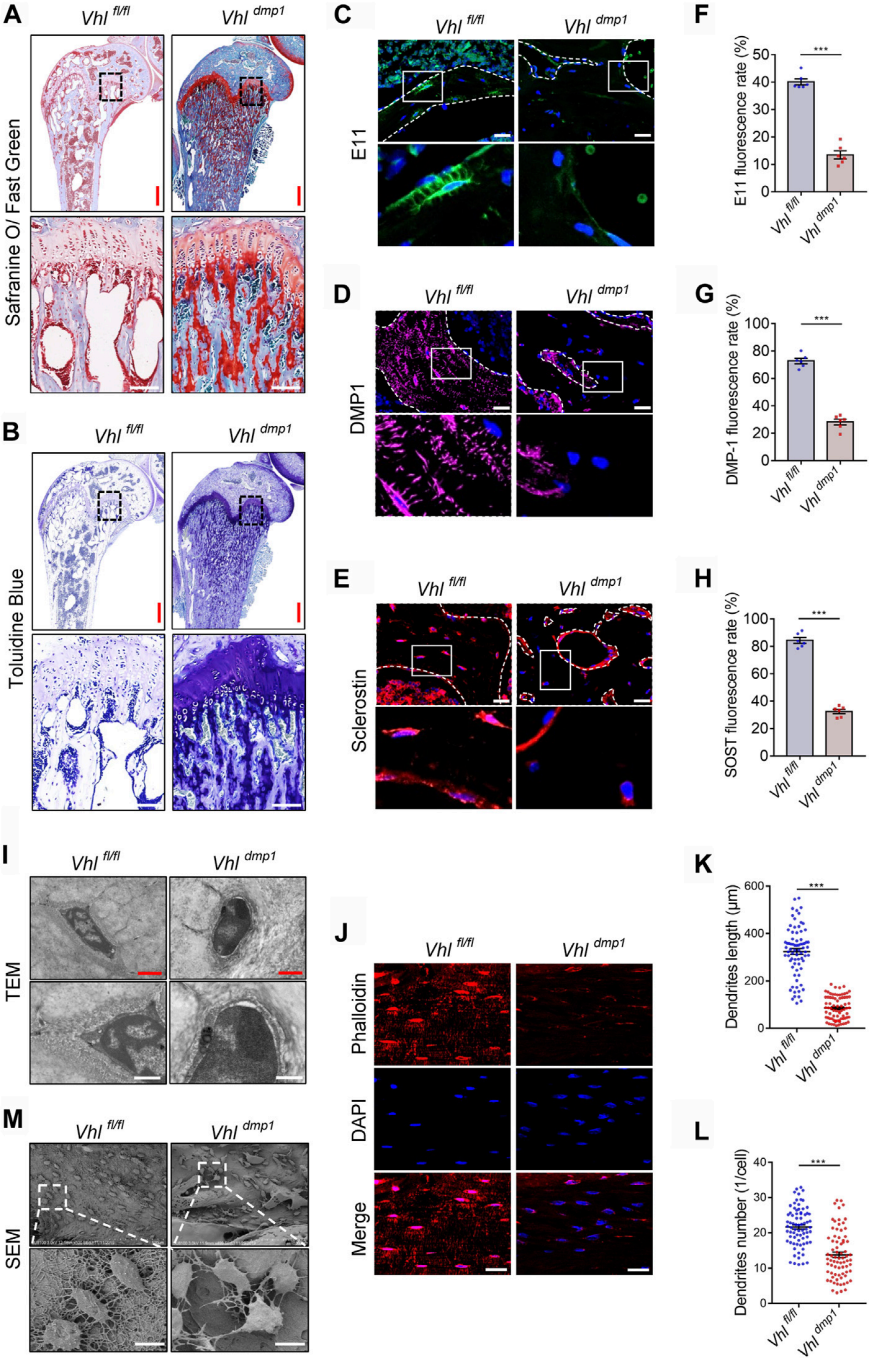
Constitutive activation of HIF-1α impairs osteocytes terminal differentiation *in vivo*. **(A,B)** Representative images of Safranin O and toluidine blue staining in the femurs of 2-month-old *Vhl* ^*f/f*^ *-Dmp-1-Cre* ^*+*^ and *Vhl* ^*f/f*^ *-Dmp-1-Cre* ^*−/−*^ male mice. Red and white scale bars represent 500 and 100 μm, respectively. **(C–E)** Immunostaining of E11 **(C)**, DMP-1 **(D)**, and sclerostin **(E)** in femur sections of 2-month-old *Vhl* ^*f/f*^ *-Dmp-1-Cre* ^*+*^ and *Vhl* ^*f/f*^ *-Dmp-1-Cre* ^*−/−*^ male mice. Scale bar represents 100 μm. **(F–H)** Bar charts showing the percentages of E11- **(F)**, DMP-1- **(G)**, and sclerostin-positive **(H)** cells shown in **(C–E)**. **(I)** Representative transmission electron microscopy (TEM) images showing the ultrastructure of osteocytes. Red and white scale bars represent 2 and 1 μm, respectively. **(J)** Texas red-X-conjugated phalloidin and 4′,6-diamidino-2-phenylindole (DAPI) staining of decalcified femoral cortices of 2-month-old *Vhl* ^*f/f*^ *-Dmp-1-Cre* ^*+*^ and *Vhl* ^*f/f*^ *-Dmp-1-Cre* ^*−/−*^ male mice. Scale bars represent 100 μm. **(K,L)** Bar graphs showing the dendrites length **(K)** and dendrites number **(L)** of phalloidin-positive osteocytes depicted in **(J)**. **(M)** Representative scanning electron microscopy (SEM) images showing the ultrastructure of osteocytes. Scale bar represents 10 μm ***p < 0.001. *p*-values were determined using two-tailed *t*-tests.

The impaired maturation of osteocytes can cause defects in mineralization (Pereira et al., 2019). Safranine O and toluidine blue are stains for proteoglycans. Impaired osteocyte terminal differentiation leads to the excessive accumulation of proteoglycan-rich immature bone, which is therefore positively stained by Safranin O and toluidine blue (Jia et al., 2013). We found that the majority of cancellous tissue in the subchondral bone of CA-HIF-1α mice stained positive for Safranine O and toluidine blue (Figures 3A,B), Contrary, a less staining density of Safranine O and toluidine blue was found in cancellous tissue of the subchondral bone of *Hif-1α* ^−/-^ mice than controls (Supplementary Figure S4). Which indicating that the osteocytes in CA-HIF-1α mice were immature.

Osteocytes are the long-lived descendants of terminally differentiated osteoblasts, which become embedded in calcified bone. (Bonewald, 2011). Next, we examined the effects of CA-HIF-1α on osteocytes terminal differentiation. Immunostaining revealed a decrease in the percentages of cells positive for E11, DMP-1, and SOST expression in CA-HIF-1α osteocytes compared to the controls (Figures 3C−H). Whereas *Hif-1α* deletion significantly increased the percentages (Supplementary Figure S5). Furthermore, examination of bone cell ultrastructure via transmission electron microscopy (TEM) revealed that a larger quantity of osteoid encased the osteocytes in CA-HIF-1α mice than in control mice (Figure 3I). Instead, osteocyte-specific *Hif-1α* deletion resulted in a higher level of mineralized matrix encased the osteocytes than in controls (Supplementary Figure S6A). Taken together, these results indicate that CA-HIF-1α causes significant impairments in the terminal differentiation and maturation of osteocytes.

Mature osteocytes reside within lacunae in the mineralized bone matrix, and use their dendritic processes extend through the canaliculi to form the osteocyte lacuna-canalicular network (Dallas et al., 2013). The staining of decalcified femoral cortices with Texas red-X-conjugated phalloidin revealed that the dendrites of osteocytes were dramatically shorter or absent in the CA-HIF-1α mice compared to the controls (Figures 3J–L). Osteocyte morphology and the lacuna-canalicular network detected via scanning electron microscopy (SEM) revealed that the osteocytes of CA-HIF-1α mice had no obvious axial arrangement in the cortical bone and the interconnections between osteocytes were sparse, with significant reductions in dendrite number and length compared to the controls (Figure 3M). In contrast, the interconnections between *Hif-1α* deficient osteocytes were dense, with significant inductions in dendrite number and length compared to the controls (Supplementary Figures S6B–E). Taken together, the disrupt integrity of the osteocyte/canalicular network in CA-HIF-1α mice further confirmed the impairments in the terminal differentiation and maturation of osteocytes.

### Osteocyte-specific HIF-1α Regulates Osteocyte Terminal Differentiation *in vitro*

**FIGURE 4:**
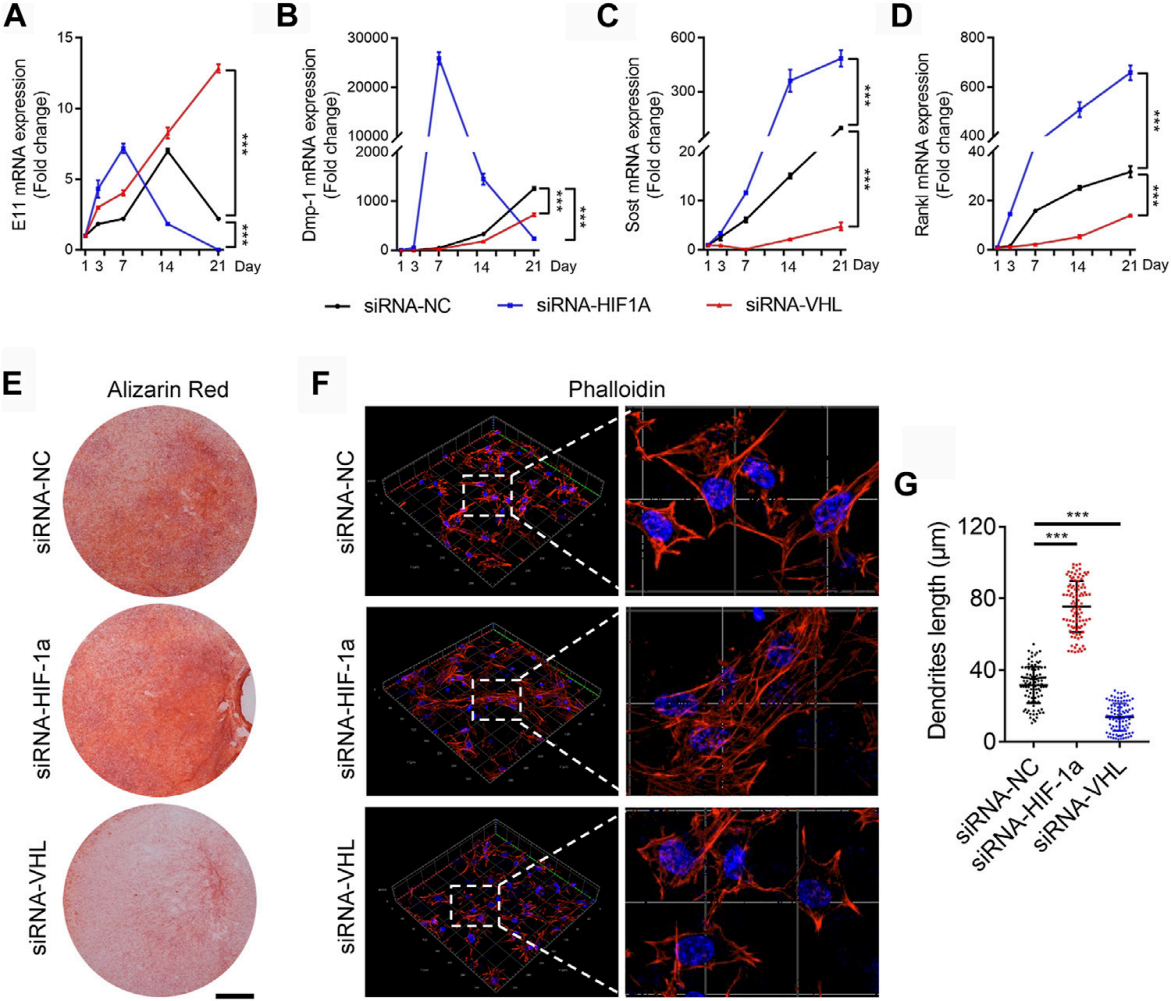
HIF-1α activation disrupts the terminal differentiation of osteocytes *in vitro*. **(A–D)** Time courses of E11 **(A)**, DMP-1 **(B)**, sclerostin (SOST) **(C)**, and RANKL **(D)** mRNA expression in IDG-SW3 cells after osteoblastic induction with or without *Vhl*-or *Hif-1α*-small interfering RNA (siRNA) transfection. **(E)** Representative images of Alizarin red staining in IDG-SW3 cells after 14 days of osteoblastic induction. Scale bar represents 5 mm. **(F)** Three-dimensional images of Texas red-X-conjugated phalloidin staining of IDG-SW3 cells transfected with or without *Vhl* or *Hif-1α*-siRNA. Scale bar represents 20 μm. **(G)** Bar graphs showing the dendrite length of phalloidin-positive osteocytes depicted in **(F)**. ***p < 0.001. *p*-values were determined using two-way ANOVA in **(A–D)** and two-tailed *t*-tests in **(G)**.

We next sought to detect the governing role of HIF-1α in osteocyte differentiation and maturation *in vitro*. DMP-1 is a mineralizing osteocyte selective marker that plays a role in matrix mineralization (Gluhak-Heinrich et al., 2003; Bonewald, 2011). To investigate whether the HIF-1α pathway mediates the process of osteocyte differentiation and mineralization *in vitro*, we induced osteocytic differentiation in the immortomouse/DMP-1-GFP-derived bone cell line (IDG-SW3) (Woo et al., 2011). HIF-1α activation or inhibition was induced in IDG-SWG cells upon transfection with *Vhl*- or *Hif-1α*-specific small interfering RNA (siRNA), respectively. As shown in Figures 4A–C, Supplementary Figure S7, *Vhl* silencing caused a delay, whereas *Hif-1α* knockdown resulted an advance in the expression of the osteoid osteocyte marker *E11*, the mineralizing osteocyte marker *Dmp-1*, and the mature osteocyte marker *sclerostin (Sost)* during osteocytic differentiation of IDG-SW3 cells. Since mature osteocytes are the main producers of receptor activator of nuclear factor kappa-Β ligand (RANKL) (Xiong et al., 2015), a decrease and increase in *Rankl* expression was observed in *Vhl*- and *Hif-1α* silenced cells, respectively (Figure 4D). In addition, extracellular matrix mineralization detected via Alizarin red S staining and dendrites formation detected by Phalloidin staining, provided further evidence for impaired osteocyte terminal differentiation in IDG-SW3 cells with *Hif-1α* activation or silencing (Figures 4E–G).

### Osteocyte-specific HIF-1α activation drives osteoblastic differentiation of MSCs and impairs osteoclastic differentiation of BMMs

**FIGURE 5:**
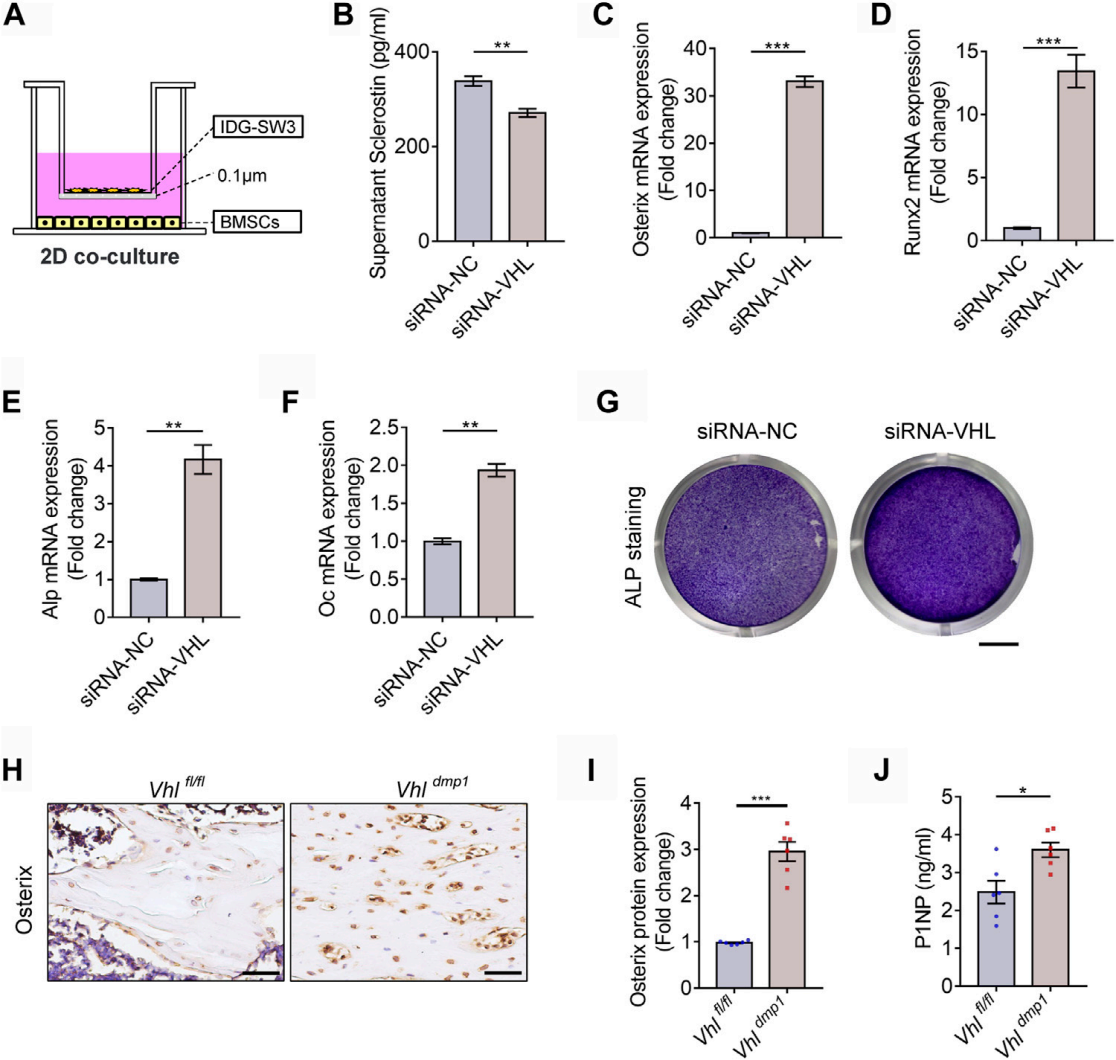
Constitutive activation of the HIF-1α in osteocytes drives osteoblastic differentiation of bone marrow mesenchymal stem cells (BMSCs). **(A)** Schematic diagram depicting the two-dimensional co-culture (2D co-culture) system of BMSCs and IDG-SW3 cells with or without VHL-siRNA transfection in Transwell plates. **(B)** Bar graph showing the sclerostin concentrations in the supernatants of osteocytes with or without VHL-siRNA transfection depicted in **(A)**. **(C–F)** mRNA expression levels of Osterix **(D)**, RUNX2 **(E)**, ALP **(F)**, and osteocalcin (OC) **(G)** in BMSCs cultured in the system depicted in **(A)**. **(G)** Alkaline phosphatase (ALP) staining of BMSCs cultured in the system depicted in **(A)**. Scale bar represents 4 mm. **(H)** Osterix immunohistochemical staining in femur sections of 2-month-old *Vhl* ^*f/f*^ *-Dmp-1-Cre* ^*+*^ and *Vhl* ^*f/f*^ *-Dmp-1-Cre* ^*−/−*^ male mice. Scale bar represents 50 μm. **(I)** Bar graph represents the relative protein expression of Osterix in **(H)**. **(J)** Bar graph showing the serum concentrations of procollagen type 1 N-terminal propeptide (P1NP) in *Vhl* ^*f/f*^ *-Dmp-1-Cre* ^*+*^ and *Vhl* ^*f/f*^ *-Dmp-1-Cre* ^*−/−*^ mice. *p < 0.05, **p < 0.01, ***p < 0.001. *p*-values were determined using two-tailed *t*-tests.

Osteocytes are multifunctional cells that play key regulatory roles such as the governing of bone formation by osteoblasts and bone resorption by osteoclasts. Mature osteocytes are the main producers of SOST and RANKL, which are key factors that regulate osteogenesis in osteoblast progenitors and osteoclastic differentiation of BMMs, respectively. In addition to abnormal bone formation, our results also revealed that CA-HIF-1α mice exhibited reduced *Sost* and *Rankl* expression. Which indicated that CA-HIF-1α in osteocytes may drive excessive bone formation by disrupting osteoblastic differentiation of MSCs and osteoclastic differentiation of BMMs, respectively. To test it, IDG-SW3 cells (with or without *Vhl*-siRNA transfection) were co-cultured with bone marrow MSCs in Transwell plates with two-dimensional (2D) system (Figure 5A). As shown in Figure 5B, Sclerostin secretion was significantly lower in co-cultures containing *Vhl*-silenced IDG-SW3 cells. Several osteogenesis-related genes were upregulated in BMSCs co-cultured with VHL-silenced IDG-SW3 cells, including *Osterix* (Figure 5C), *Runx2* (Figure 5D), *alkaline phosphatase* (*Alp*) (Figure 5E), and *Osteocalcin* (*OC*) (Figure 5F). Moreover, the density of ALP staining was higher in CA-HIF-1α/IDG-SW3 co-cultures compared to the control (Figure 5G). This suggests that HIF-1α activation in osteocytes facilitates MSCs osteogenesis. In accordance with our *in vitro* co-culture results, *Osterix* expression was upregulated in osteoblast progenitors on the trabecular bone surface of CA-HIF-1α mice, as confirmed by immunohistochemical staining (Figures 5H,I). Moreover, serum levels of the bone formation marker procollagen type 1 N-terminal pro-peptide (P1NP) were also significantly higher in CA-HIF-1α mice compared to control mice (Figure 5J). Collectively, these findings suggest that CA-HIF-1α in osteocytes drives osteogenesis of MSCs.

**FIGURE 6:**
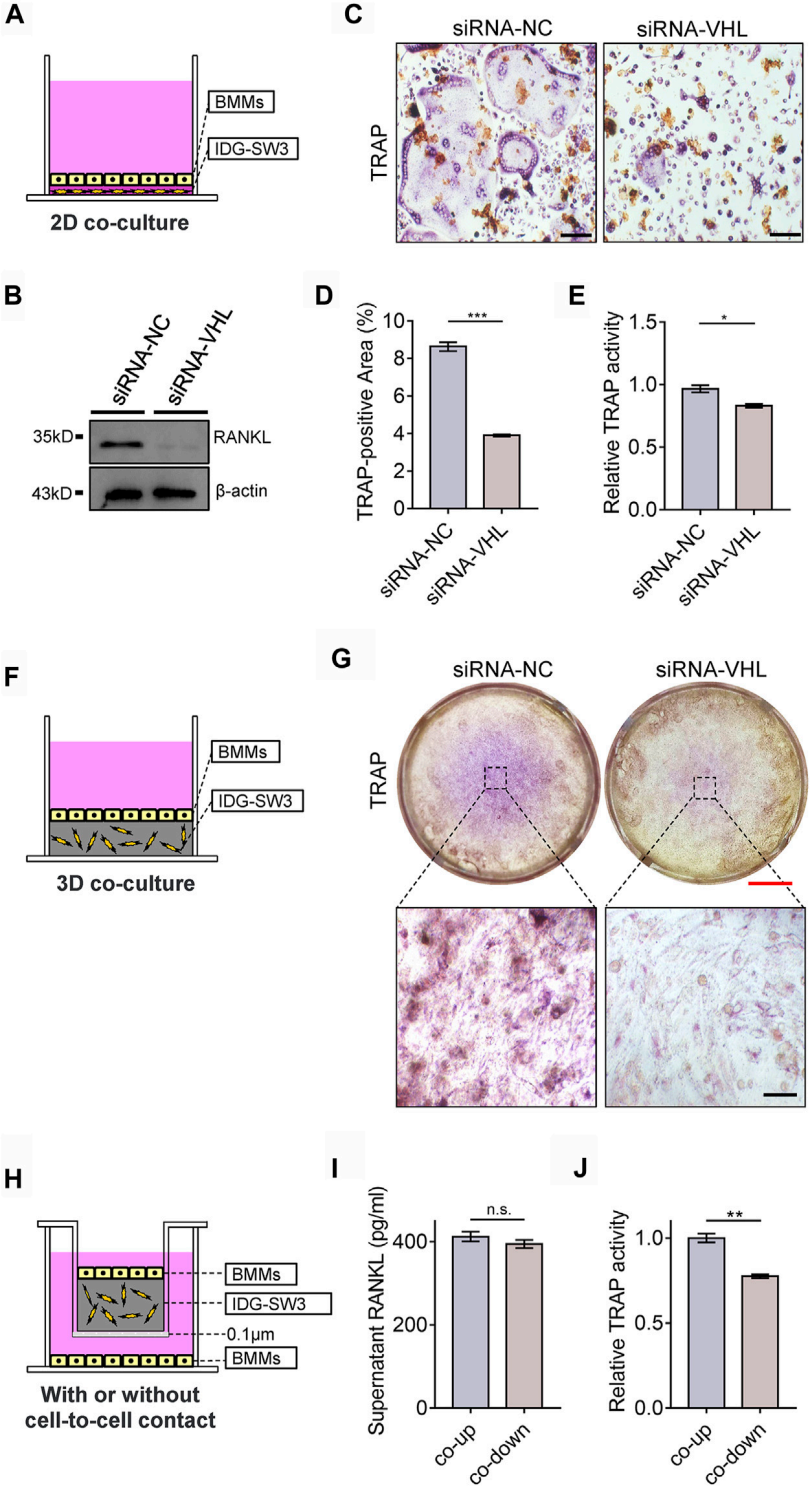
Constitutive activation of the HIF-1α in osteocytes impairs osteoclastic differentiation of bone marrow monocytes (BMMs). **(A)** Schematic diagram depicting the two-dimensional system used for the co-culture of BMMs and IDG-SW3 cells with or without VHL-siRNA transfection in Transwell plates. **(B)** Western blot showing RANKL expression in IDG-SW3 cells cultures in the system depicted in **(A)**. **(C)** Representative images of tartrate-resistant acid phosphatase (TRAP) staining of BMMs cultured in the system depicted in (a). Scale bar represents 50 μm. **(D)** Bar graph showing the percentage of TRAP-positive area calculated from the images shown in **(C)**. **(E)** Bar graph showing the relative TRAP activity of BMMs cultured in the system depicted in **(A)**. **(F)** Schematic diagram depicting the three-dimensional system used to co-culture BMMs and IDG-SW3 cells with or without VHL-siRNA transfection in Transwell plates. **(G)** Representative images of TRAP staining of BMMs cultured in the system depicted in **(F)**. Red and black scale bar represents 2 mm and 50 μm, respectively. **(H)** Schematic diagram showing IDG-SW3 cells co-cultured with BMSCs (co-up) with or without (co-down) cell-to-cell contact. **(I)** Bar graph showing the RANKL concentrations in the supernatants of co-up and co-down cultures depicted in **(H)**. **(J)** Bar graph showing the relative TRAP activity of BMMs cultured in the co-up and co-down systems depicted in **(H)**. *p < 0.05, **p < 0.01, ***p < 0.001. *p*-values were determined using two-tailed *t*-tests.

To investigate the role of osteocytic HIF-1α activation in BMM osteoclastic differentiation, we set up an *in vitro* 2D co-culture system containing BMMs and IDG-SW3 cells with or without VHL-siRNA transfection (Figure 6A). RANKL expression was dramatically lower in 2D co-cultures containing *Vhl*-siRNA-transfected IDG-SW3 cells compared to the control (Figure 6B). Furthermore, *Vhl* silencing in IDG-SW3 cells caused impairments in osteoclastic differentiation of BMMs, as shown by the reduction in multinuclear osteoclast formation and decreased tartrate-resistant acid phosphatase (TRAP) activity compared to the controls (Figures 6C–E). Next, we examined the effects of CA-HIF-1α on osteoclastic differentiation using a three-dimensional (3D) system, in which BMMs were co-cultured with IDG-SW3 cells with or without *Vhl*-siRNA transfection (Figure 6F); TRAP staining showed that osteoclastic differentiation was dampened in BMMs cultured with *Vhl*-siRNA-transfected IDG-SW3 cells compared to the control (Figure 6G).

RANKL is initially synthesized as a membrane-bound protein that can be cleaved into a soluble form (Xiong et al., 2018). The membrane-bound form of RANKL, as opposed to its soluble form, contributes to physical bone remodeling and ovariectomy-induced bone loss by promoting the formation of osteoclasts (Asano et al., 2019). To explore this relationship *in vitro*, we cultured BMMs and IDG-SW3 cells in a 3D co-culture system, in which the IDG-SW3 osteocytes and BMMs were either cultured proximally with cell-to-cell contact (co-up) or cultured in the same well and medium without cell-to-cell contact (co-down) (Figure 6H). Our results further suggest that the membrane-bound form of RANKL promotes osteoclastic differentiation in BMMs, as the soluble RANKL concentrations in the medium were comparable in both the co-up and co-down cultures (Figure 6I), whereas TRAP activity was significantly higher in BMMs cultured with cell-to-cell contact with IDG-SW3 cells compared to those cultured without IDG-SW3 cell contact (Figure 6J). Taken together, these results indicate that CA-HIF-1α in osteocytes inhibits osteoclastic differentiation of BMMs.

## Discussion

The VHL/HIF system is highly active in skeletal tissue. Deletion of PHD or VHL stabilizes HIF-1α, which causes an increase in bone mass by disrupting the balance between bone formation by osteoblasts and bone resorption by osteoclasts (Zhang et al., 2014; Wu et al., 2015; Kang et al., 2017; Loots et al., 2018). Furthermore, activation of the HIF-1α pathway using hypoxia-mimicking agents such as deferoxamine and dimethyloxalylglycine is a promising treatment for bone diseases such as osteoporosis and skeletal fractures (Zhao et al., 2012; Peng et al., 2014; Jia et al., 2016; Kang et al., 2016). However, CA-HIF-1α induced by VHL deletion or PHD1/2/3 inactivation causes excessive bone formation and polycythemia (Wang et al., 2007; Wu et al., 2015) as well as disrupts the integrity of the osteocyte/canalicular network (Zuo et al., 2015). Therefore, fully investigations into the effects of HIF-1α activation on bone micro-structure and remodeling are needed before clinically applying hypoxia-mimicking agents as treatments for skeletal diseases.

Osteocytes are the most abundant cells within bone tissue, constituting 90–95% of all bone cells (Schaffler & Kennedy, 2012). Osteocyte-specific *Vhl* deletion results in HIF-1α activation and a high bone mass phenotype and hematopoietic defects (Loots et al., 2018). Herein we further observed that osteocyte-specific *Hif-1α* deletion leaded to decreased bone mass. In addition, we found that *Vhl* deletion caused impairments in the elastic modulus and nanohardness of trabecular bone, suggesting that CA-HIF-1α in osteocytes leads to compromised bone material properties.

Relationships between bone material properties such as the degree of mineralization, crystallinity, and collagen characteristics and parameters such as the local modulus and nanohardness have been established in animal and human bones (Smith et al., 2010). Abnormal mineralization and impaired collagen networks typically lead to compromised bone material properties (Albert et al., 2013). In the present study, we found that CA-HIF-1α in osteocytes significantly increased bone collagen content, whereas the collagen fibers were more disorganized compared to the control. In addition, the mineralization levels of cortical and trabecular bone were significantly lower in CA-HIF-1α mice compared to the controls. Instead, osteocyte-specific *Hif-1α* deletion resulted in too fast bone mineralization and caused a significant decrease in bone collagen accumulation. These factors might have contributed to the impaired bone material properties of the CA-HIF-1α mice.

Mature osteoblasts become embedded within the bone matrix and differentiate into osteocytes. Impairments in the maturation and differentiation of osteoblasts and osteocytes contribute to mineralization defects (Pereira et al., 2019). In this study, we found that the majority of cancellous tissue in the subchondral bone of CA-HIF-1α mice was stained by Safranin O and toluidine blue, indicating the presence of proteoglycans and therefore, immature bone tissue. Furthermore, following examination of the bone cell ultrastructure via TEM, we found that osteocyte bodies were larger in CA-HIF-1α mice than in control mice, more osteoid components were distributed outside the cells, and large quantities of collagen with varying levels of calcification surrounded the osteocytes. This indicated that the majority of the osteocytes in CA-HIF-1α mice were immature. Contrary, a less staining density of Safranine O and toluidine blue was found in cancellous tissue of the subchondral bone and a higher level of mineralized matrix encased the osteocytes in *Hif-1α* ^−/-^ mice than in controls.

Osteocytes are derived from mature osteoblasts; however, unlike osteoblast differentiation from MSCs, the molecular mechanisms underlying osteocyte differentiation and maturation are largely unknown (Zeng et al., 2017). Here, we found that HIF-1α activation caused decreases in the expression of the osteoid osteocyte marker E11, the mineralizing osteocyte marker *Dmp-1*, and the mature osteocyte marker *Sost*. To further explore the regulatory role of HIF-1α in osteocyte differentiation, we performed *in vitro* experiments using IDG-SW3 osteocytes. The IDG-SW3 cell line undergoes osteoblast-to-osteocyte differentiation by transitioning through the mature osteoblast, pre-osteocyte, and late osteocyte stages (Woo et al., 2011). We found that HIF-1α activation via VHL silencing in IDG-SW3 cells caused decreased expression of E11, *Dmp-1*, and *Sost* as well as impaired extracellular matrix mineralization during terminal osteocyte differentiation. whereas *Hif-1α* knockdown resulted an adverse effect on E11, *Dmp-1*, and *Sost* expression. These results indicate that VHL silencing, thus CA-HIF-1α, inhibits osteocyte terminal differentiation and maturation.

Osteocytes reside within the lacunae of the mineralized bone matrix, from which the dendritic processes of the osteocytes extend through the canaliculi to form the osteocyte lacuna-canalicular network. This network connects the osteocytes to cells on the bone surface and the vascular system, thereby providing oxygen and nutrients that maintain osteocyte viability in the enclosed bone matrix environment (Dallas et al., 2013). Our previous study revealed that activation of the HIF-1α pathway in mature osteoblasts impairs osteoblast terminal differentiation and disrupts the integrity of the osteocyte/canalicular network (Zuo et al., 2015). Similarly, our present work revealed that the dendrites of osteocytes were dramatically shorter or absent in CA-HIF-1α mice. Moreover, the osteocytes of CA-HIF-1α mice did not exhibit obvious axial arrangements in the cortical bone, the interconnections between the osteocytes were sparse, and the number and length of dendrites were significantly reduced in CA-HIF-1α osteocytes compared to the control. Similarly, IDG-SW3 osteocytes with constitutively active HIF-1α induced by *Vhl* siRNA silencing exhibited fewer and shorter dendrites compared to the control. In contrast, the interconnections between *Hif-1α* deficient osteocytes were dense, with significant inductions in dendrite number and length compared to the controls. These results suggest that HIF-1α activation in osteocytes disrupts the integrity of the osteocyte/canalicular network.

Bone homeostasis is continually maintained through the process of bone remodeling. As secretory cells, osteocytes participate in the regulation of bone homeostasis, bone mineralization, and bone remodeling by secreting osteogenic and osteolytic factors such as SOST, Dickkopf 1, RANKL, and osteoprotegerin (Poole et al., 2005; Nakashima et al., 2011). In accordance with previous studies (Loots et al., 2018), we found that *Sost* expression levels were lower in the osteocytes of CA-HIF-1α mice compared to the controls. However, it is still unclear whether HIF-1α activation in osteocytes directly regulates osteogenesis in osteoblast progenitors. By co-culturing osteocyte IDG-SW3 and BMSCs *in vitro*, we discovered that constitutive HIF-1α activation in osteocytes promotes osteoblastic differentiation in MSCs. Furthermore, we found that the number of Osterix-positive osteoblast progenitors on the trabecular bone surface was significantly higher in CA-HIF-1α mice.

In addition to reduced SOST expression, RANKL expression was impaired in osteocytes of CA-HIF-1α mice, as well as in VHL-silenced IDG-SW3 cells during terminal osteocyte differentiation. Moreover, CA-HIF-1α mice exhibited a decreased osteolytic phenotype, as indicated by the reduced serum levels of C-terminal telopeptide of type I collagen (CTX-I), a marker for bone resorption. With our *in vitro* co-culture system, we obtained the first evidence for the direct regulation of osteoclastic differentiation of BMMs via osteocytic HIF-1α pathway activation. Our results support previous data suggesting that osteocytes mediate osteoclastic differentiation of BMMs via membrane-bound RANKL and cell-to-cell contact, rather than soluble RANKL.

Although these results clearly revealed a critical role of osteocytic HIF-1α for bone formation, bone mineralization, collagen fiber formation, osteocyte/canalicular network, and osteocyte terminal differentiation, whether HIF-1α activation was involved in these disrupted phenotypes of bone micro-structure and remodeling observed in osteocytic Vhl-deficient mice still need for further illustration. In addition, the exact role of osteocytic HIF-2α in bone micro-structure and remmodeling, especially in impaired bone micro-structure and remodeling induced by *Vhl* deletion still needs further investigation. Although HIF-2α has been reported as a negative regulator of osteoblastogenesis and bone ma**ss** accrual (Merceron et al., 2019).

In conclusion, these data demonstrated that CA-HIF-1α in mineralizing osteocytes resulted in impaired bone micro-structures and abnormal bone remodeling, which are result from disrupted osteocyte terminal differentiation. These adverse effects suggest that moderate HIF-1α activation should be addressed before the clinical application of therapeutics involving alterations to the HIF-1α signaling pathway to treat bone diseases.

## Data Availability

The original contributions presented in the study are included in the article/Supplementary Material, further inquiries can be directed to the corresponding authors.
