# Supplementary material for: Osteocytic HIF-1α Pathway Manipulates Bone Micro-structure and Remodeling via Regulating Osteocyte Terminal Differentiation
Source: Front Cell Dev Biol. 2022 Jan 18;9:721561. doi: 10.3389/fcell.2021.721561 (PMC8804240; doi:10.3389/fcell.2021.721561)
Supplement: Supplementary file 1 [file Image1.PDF]

Supplementary files

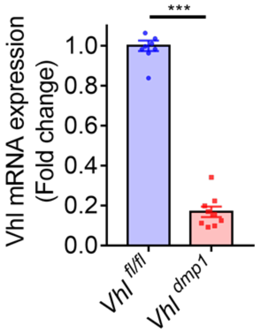

Fig. S1. ***Vhl*** **expression was significantly decreased in** ***Vhl***$^{fl/fl}$***-Dmp1-Cre***$^{-/-}$ **mice.**

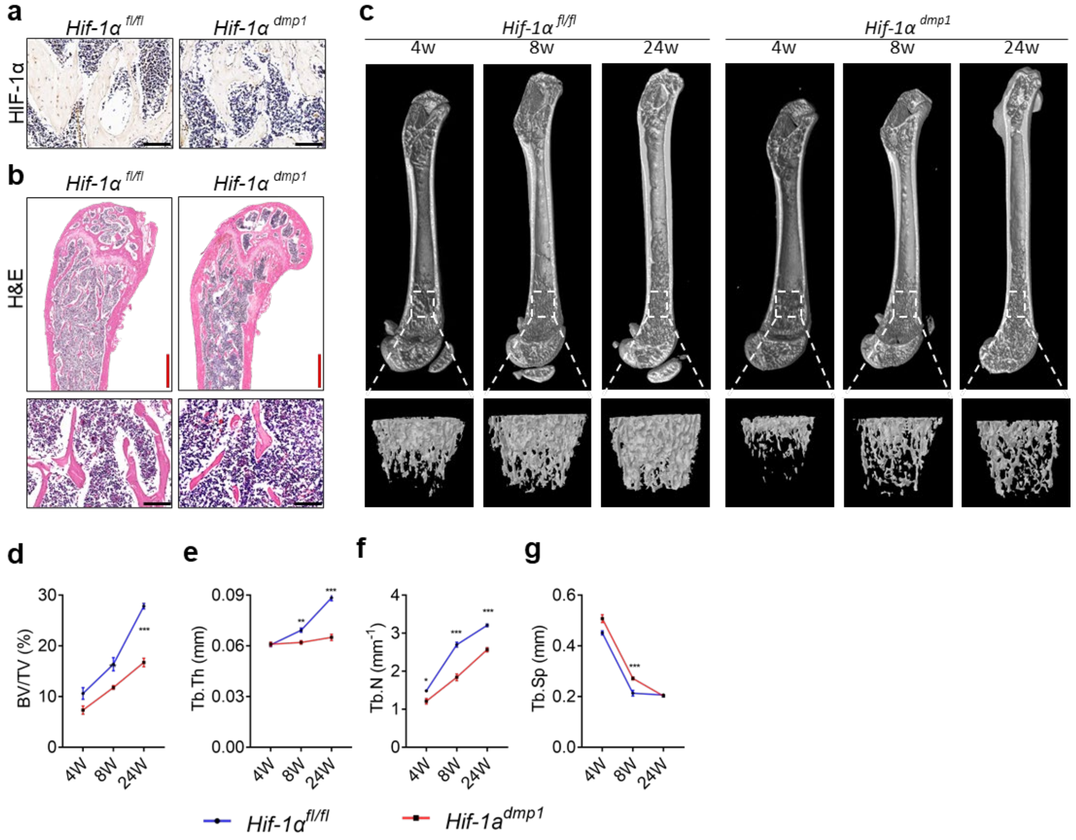

Fig. S2. Osteocyte-specific ***Hif-1α*** **deletion resulted in decreased bone formation.** (a) Representative images of immunohistochemical staining of HIF-1α in the femurs of 2-month-old *Hif-1α*$^{f/f}$*-Dmp-1-Cre*$^{+}$ and *Hif-1α*$^{f/f}$*-Dmp-1-Cre*$^{-/-}$ male mice. Scale bars represent 100 μm. (b) Representative images showing hematoxylin and eosin (H&E) staining of femurs of 2-month-old *Hif-1α*$^{f/f}$*-Dmp-1-Cre*$^{+}$ and *Hif-1α*$^{f/f}$*-Dmp-1-Cre*$^{-/-}$ male mice. Red and black scale bars represent 500 and 100 μm, respectively. (c) Representative micro-computed tomography (micro-CT) images of femurs and the corresponding trabecular bone from the femoral metaphysis of 4-, 8-, and 24-week-old

Hif-1α^{f/f}-Dmp-1-Cre^{+} and *Hif-1α^{f/f}-Dmp-1-Cre^{−/−}* male mice. (d–g) The trabecular bone volume (BV/TV) (d), trabecular number (Tb.N) (e), trabecular separation (Tb.Sp) (f), and trabecular thickness (Tb.Th) (g) parameters of 4-, 8-, and 24-week-old *Hif-1α^{f/f}-Dmp-1-Cre^{+}* and *Hif-1α^{f/f}-Dmp-1-Cre^{−/−}* male mice were determined via micro-CT analysis. **P* < 0.05, ***P* < 0.01, ****P* < 0.001. *P*-values were determined using two-way ANOVA.

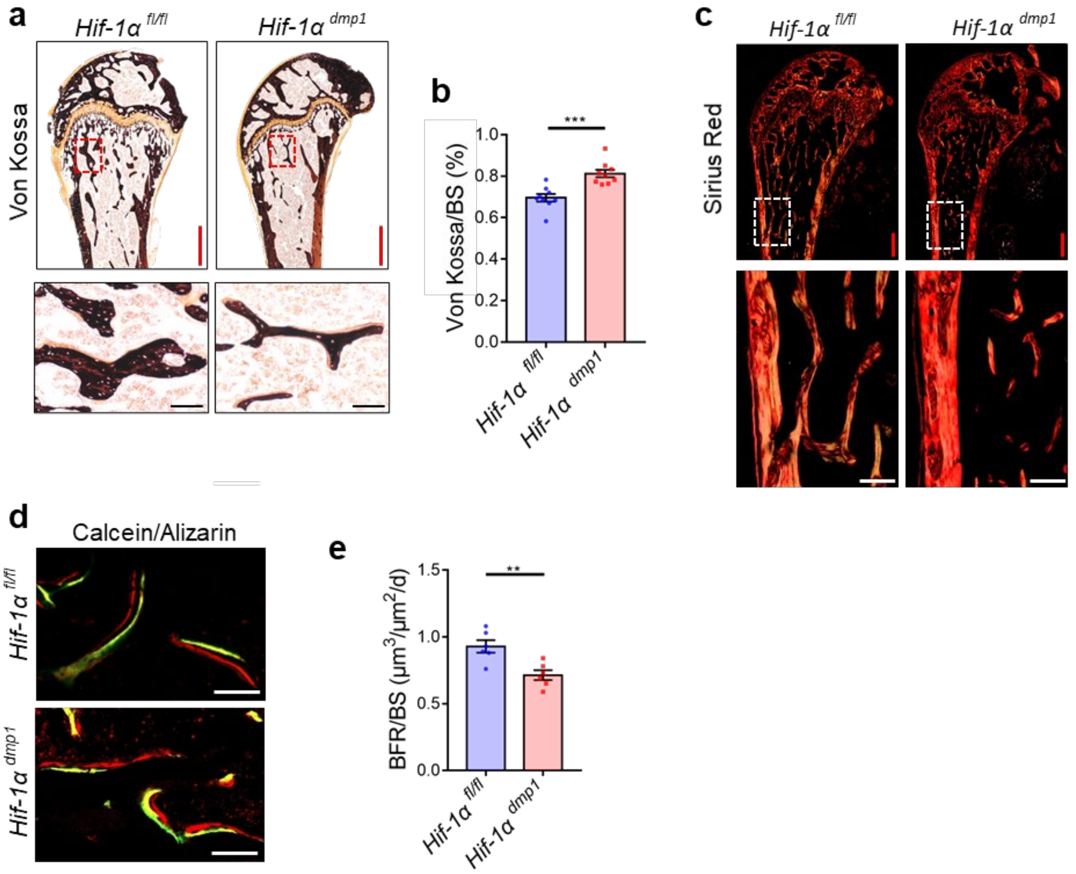

Fig. S3. Osteocyte-specific *Hif-1α* deletion resulted in abnormal mineralization and disrupted collagen fiber formation in bone. (a, b) Von Kossa staining and bar graph showing the proportional Von Kossa-positive surface area relative to total bone surface area (Von Kossa/BS) in the femurs of 2-month-old *Hif-1α^{f/f}-Dmp-1-Cre^{+}* and *Hif-1α^{f/f}-Dmp-1-Cre^{−/−}* male mice. Red and black scale bars represent 500 and 100 μm, respectively. (c) Sirius Red staining of the femurs of 2-month-old *Hif-1α^{f/f}-Dmp-1-Cre^{+}* and *Hif-1α^{f/f}-Dmp-1-Cre^{−/−}* male mice. Red and white scale bars represent 500 and 100 μm, respectively. (d, e) Representative images of double calcein/alizarin labeling of the femurs of 2-month-old *Hif-1α^{f/f}-Dmp-1-Cre^{+}* and *Hif-1α^{f/f}-Dmp-1-Cre^{−/−}* male mice (d) used to calculate the proportional mineral surface area over total bone surface area (MS/BS) (e). Scale bars represent 100 μm. ***P* < 0.01, ****P* < 0.001. *P*-values were determined using two-tailed *t*-tests.

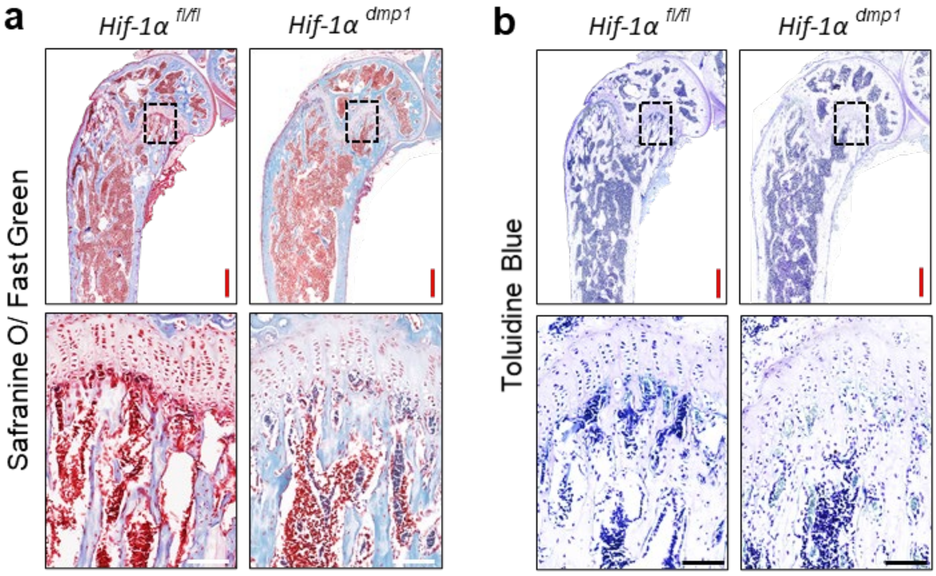

Fig. S4. Osteocyte-specific *Hif-1α* deletion advanced osteocytes maturation *in vivo*. (a & b) Representative images of Safranin O and toluidine blue staining in the femurs of 2-month-old *Hif-1α*$^{f/f}$*-Dmp-1-Cre*$^{+}$ and Hif-1α$^{f/f}$-Dmp-1-Cre$^{-/-}$ male mice. Red and white and black scale bars represent 500 and 100 and 100 μm, respectively.

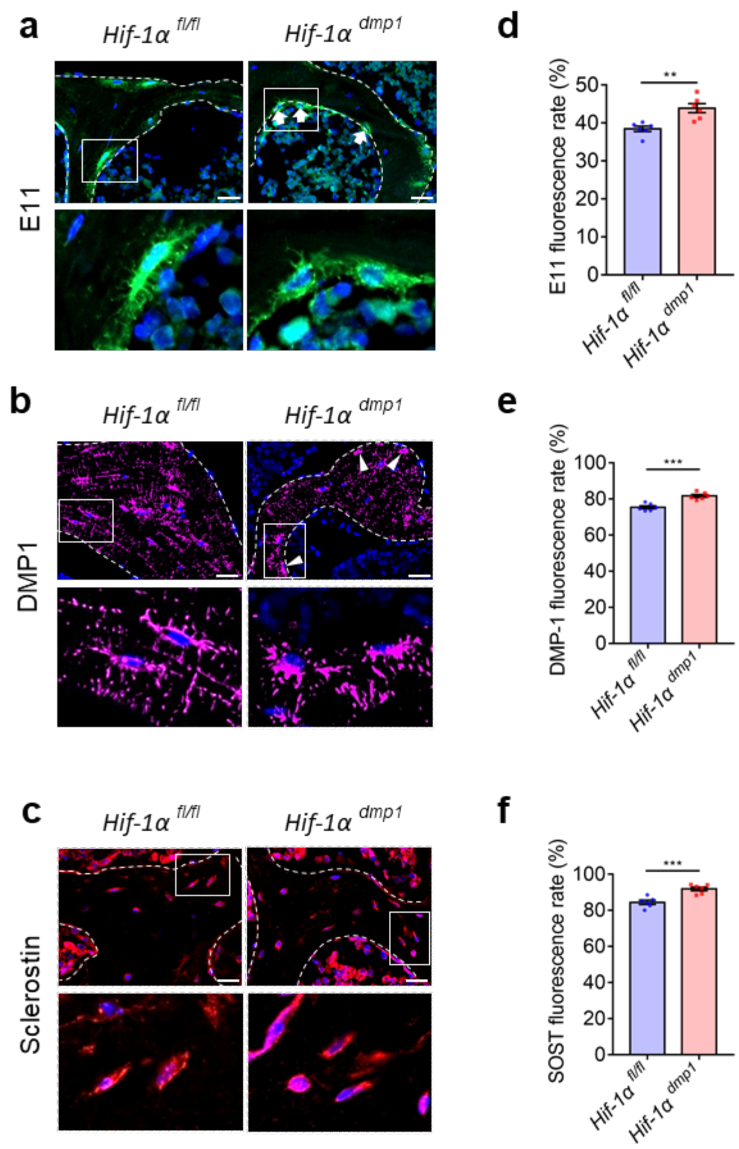

Fig. S5. Osteocyte-specific *Hif-1α* deletion advanced osteocytes terminal differentiation *in vivo*. (a–c) Immunostaining of E11 (c), DMP-1 (d), and sclerostin (e) in femur sections of 2-month-old *Vhl$^{f/f}$-Dmp-1-Cre^{+}* and *Vhl$^{f/f}$-Dmp-1-Cre$^{-/-}$* male mice. Scale bar represents 100 μm. (d–f) Bar charts showing the percentages of E11- (d), DMP-1- (e), and sclerostin-positive (f) cells shown in (a–c). $^{**}P < 0.01$, $^{***}P < 0.001$. *P*-values were determined using two-tailed *t*-tests.

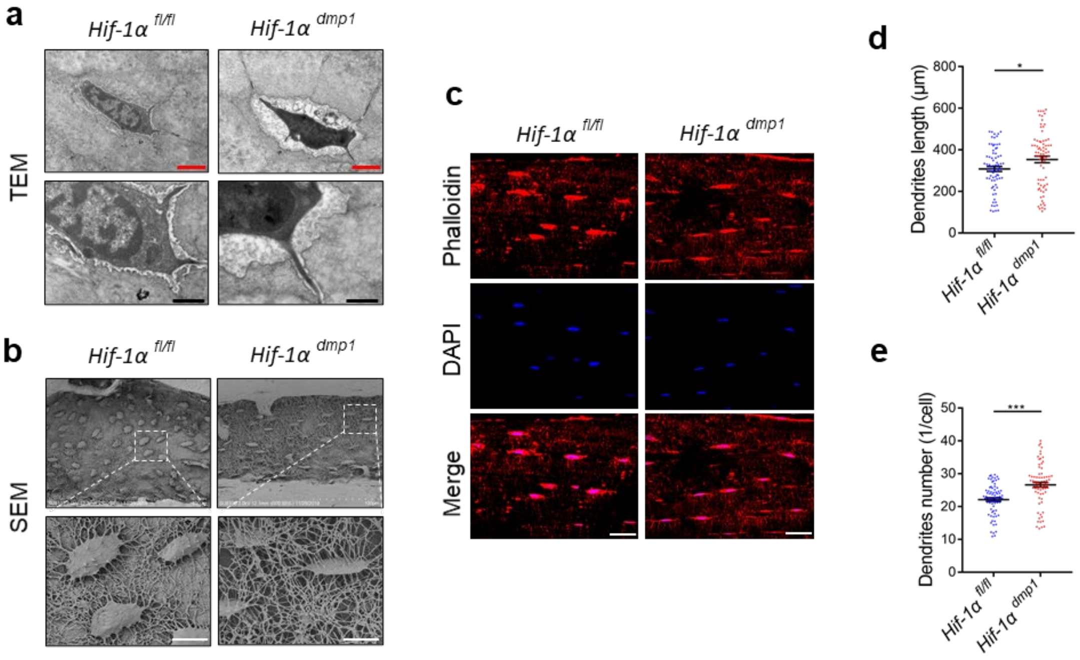

Fig. S6. Osteocyte-specific HIF-1α deletion disrupted osteocyte morphology and osteocyte/canalicular network *in vivo*. (a) Representative transmission electron microscopy (TEM) images showing the ultrastructure of osteocytes with *Hif-1α* deletion. Red and white scale bars represent 2 and 1 μm, respectively. (b) Representative scanning electron microscopy (SEM) images showing the ultrastructure of osteocytes with *Hif-1α* deletion. Scale bar represents 10 μm. (c) Texas red-X-conjugated phalloidin and 4',6-diamidino-2-phenylindole (DAPI) staining of decalcified femoral cortices of 2-month-old male mice with osteocyte-specific *Hif-1α* deletion. Scale bars represent 100 μm. (d, e) Bar graphs showing the dendrites length and dendrites number of phalloidin-positive osteocytes depicted in (c). $*P < 0.05$, $***P < 0.001$. *P*-values were determined using two-tailed *t*-tests.

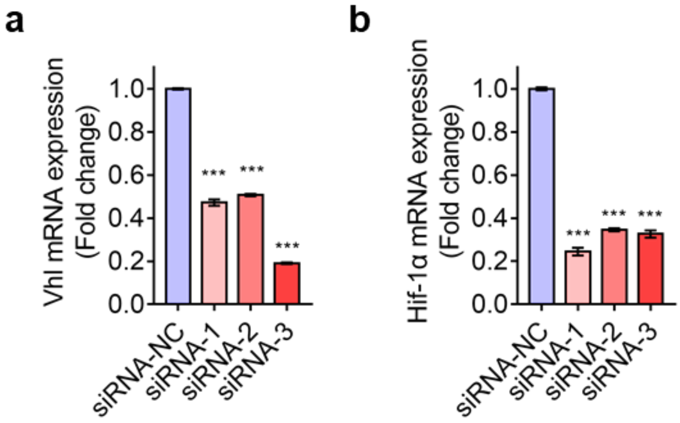

Fig. S7. mRNA expression of *Vhl* and *Hif-1α* in IDG-SW3 cells after transfected with siNRA oligonucleotides.
